# Dynamics of Multimorbidity Resilience and Health Outcomes Over Time in Community-Residing Older Adults

**DOI:** 10.1093/geroni/igaa057.3285

**Published:** 2020-12-16

**Authors:** Yingzhi Xu, Zahra Rahmaty, Eleanor McConnell, Tingzhong (Michelle) Xue, Bada Kang, Kirsten Corazzini

**Affiliations:** 1 Duke University, Durham, North Carolina, United States; 2 University of Maryland School of Nursing, Baltimore, Maryland, United States; 3 Duke University School of Nursing, Durham, North Carolina, United States

## Abstract

Multimorbidity resilience may mitigate the adverse effects of multiple chronic diseases on older adults’ health. Wister et al.’s (2018) multimorbidity resilience index was developed and tested in a cross-sectional sample of older adults in Canada. Building on these findings, we examined the reciprocal relationships of resilience on outcomes to test these potentially mitigating effects in a community-based, U.S. sample of older adults over time. The study sample includes 1,054 older adults from waves 2 and 3 of the National Social Life, Health, and Aging Project (NSHAP) study (Waite et al 2020). Wister et al.’s (2018) index was mapped to NSHAP measures, and reciprocal relationships of multimorbidity resilience and health outcomes over a 5-year period was tested using structural equation modeling (SEM). Results indicated significant effects of multimorbidity resilience on self-rated physical health and pain. Interestingly, a better functional resilience at baseline conferred better self-rated physical health at follow-up, while better psychological resilience predicted lower pain level. By contrast, the influence of health outcomes on any domain of multimorbidity resilience was not detectable at all, supporting the direction of these associations from resilience to outcomes. The study systematically investigated the dynamic hypotheses between multimorbidity resilience and health outcomes. That is, whether they are determinants or consequences, or both. Our findings suggest multimorbidity resilience predicts subsequent 5-year change in health outcomes, especially self-rated physical health and pain level, but not vice versa, strengthening the evidence of the importance of resilience in the health of older adults.

